# Periscope of Renal and Urinary Pathology

**Published:** 1880-10

**Authors:** 


### Summary.

### PERISCOPE OF RENAL AND URINARY PATHOLOGY.

1. The *Cincinnati Lancet and Clinic* (Aug 7, 1880) reports a case from *Le Praticien* in the practice of M. Hardy, in which the presence of indican in the urine was regarded as important in establishing the diagnosis in a doubtful case of typhoid fever. Exactly what relation indican bears to typhoid, more than to any other continued fever, is not stated.

2. "Extirpation of the Kidney. Death on the fourth day after operation." By Fred. Lange, M.D. New York: *Med. Record*, Aug. 7, 1880.

Dr. Lange's case is one of great pathological and surgical interest. He extirpated the right kidney, after careful and repeated examinations, assisted by other medical gentlemen. A diagnosis of pyonephrosis, involving the right kidney was made. The left kidney was supposed to be in a healthy condition, mainly because, when the symptoms pointed to the conclusion that the right ureter was closed temporarily by pus and products of degeneration from the tumefied, tender and painful right kidney, the discharge of urine became suddenly normal (or very nearly so) as to quality and did not lessen in quantity. As Dr. Lange says: "nothing was more probable than to assume that the (right) ureter was now completely or almost completely obstructed, and that the urine was discharged from the other (left) side." The patient demanded that something "decisive" be done. Accordingly Dr. Lange "proposed an exploratory incision" with the intention of removing the diseased organ or incising it, as should seem wisest. It was thought best, upon reaching the diseased organ, to extirpate it, as it seemed to be wholly devoid of functional power, and the left kidney was still regarded as healthy. The patient rallied well from the operation, "and presented a good condition, complaining merely of pain in her back and right hip. * * * not a drop of urine was found in the bladder until her death, which occured on the fourth day, eighty-four hours after the operation."

The post mortem readily explained the immediate cause as well as the mode of death, which was in a state of profound coma. We quote Dr. Lange's language: "The left kidney, which was removed by autopsy, consists of several cystic cavities, two of which contain a fluid-like urine; one of about the size of a hen's egg was filled with thick, cheesy matter. There is no trace of tissue of the kidney left. The pelvis of the kidney is entirely obliterated and so is the ureter. Probably for a number of years no urine has been secreted from this organ. The disease which caused this degeneration might have occurred in the patient's earliest childhood, at least she could not remember having had any trouble in this side. The organ was at its normal place, and although cystic, it was only slightly increased in size and its shape was not materially changed from the normal. * * * The other kidney, the right one, which had been removed by operation, was about double the size of a normal one. In a longitudinal section it is seen that it consists essentially of two parts; one, embracing about the upper two-thirds, is solid, presenting tissue of the kidney in a state of hypertrophy, measuring from two to three centimeters in height. The uppermost edge has a very irregular surface, and here the tissue is scattered with small knot-like infiltrations, measuring from several millimeters to one centimeter in size. * * * A part of this tissue has been removed for microscopical examination. All those spots seem to be beginning abscesses. Some of them present a yellowish purulent appearance; others a more recent infiltration. The pelvis of the kidney, so far as it belongs to these upper two-thirds, presents a normal appearance. The lower third of the kidney is composed of a number of cysts, the largest being about the size of a small hen's egg. Those cysts contained urine and pus and seemed not to have any connnection with the pelvis. But a careful examination shows that there exist several narrow canals leading to the pelvis." The further description of the lower third of the right kidney shows that it was in an extreme state of cystic degeneration. The total amount of renal tissue capable of any functional duty possessed by the patient was the "upper two thirds of the right kidney, and even that was in a condition far from normal. Dr. Lange's grounds for concluding that the left kidney was healthy were certainly just and reasonable. He seems to us to have been unusually careful and painstaking in his diagnosis, and yet he was, as he says, "induced to perform an absolutely deadly operation." Dr. Lange's candor and honesty in relating this unfortunate case is worthy of all commendation.

3. "On a Filiaria reported to have come from a Man." (*Medical News and Abstract*, Sept., 1880.)

Prof. J. Leidy describes, in the "Proceedings of Acad. of Nat. Sci. of Phila. for March, 1880," a large thread worm, which had been submitted to his examination by Dr. J. J. Woodward, U. S. A. It was recently presented to the Army Medical Museum by Dr. C. L. Garnett of Buffalo with the following explanation: "During the winter of 1876 a common laborer presented himself to me for treatment, having a gleety discharge from the urethra with a burning sensation during and after micturition. Previously he had been treated for gonorrhœa, and I prescribed accordingly. The patient not improving, applied to other practitioners. In April, 1878, he came to me with a round, vivid-red worm, twenty-six inches in length, which was alive and very active in its movements, instantly coiling up like a watch spring on being touched. The patient is an illiterate man with no motive for deception. He informed me that he discovered the worm protruding from his penis and drew it out without pain or difficulty. Prof. Leidy says "if it really is a human parasite, it appears to differ from all those heretofore described, and also seems different from other known parasites; and "for the present," he proposes to "distinguish it with the name *Filiaria Restiformis*." (But how did the worm find its way into the patient's bladder and how long did it remain there before passing away?)

4. The Sphygmograph in the early diagnosis of Bright's disease. *British Med. Jour.*, Aug. 14, 1880.

We quote from Dr. John Buckley Bradbury's "Address on

Medicine," before the British Medical Association, at its late meeting, the following very interesting observations concerning the value of the sphygmograph in the diagnosis of commencing Bright's disease:

In the field of medicine to which the sphygmograph has been used, the harvest has been more abundant than in the case of the laryngoscope, chiefly owing to the researches of Burdon Sanderson having been elaborated by Galabin, Mahomed, and others. In his thesis, read in 1873, for the degree of M.D. in this University, Dr. Galabin, formerly Fellow of Trinity College, corroborated the views of Burdon Sanderson, George Johnson and others, on the use of this instrument in estimating the amount of arterial tension, as gauged by the height of the tidal or predicrotic wave of a pulse-tracing; and for the first time pointed out that high arterial tension exists in acute as well as chronic Bright's disease. The condition of high-blood pressure is of the greatest importance clinically, and should be watched and estimated as carefully as the body-temperature, as there is no doubt it will be when the sphygmograph has been further simplified. High blood-pressure gives the earliest indication of the grave series of degenerative changes throughout the body known as chronic Bright's disease, and may, if neglected, lead to disastrous results both in disease of the arteries and of the heart. High blood-pressure is the cause of all so-called heart-disease in old persons; it is very amenable to treatment, and its treatment is imperatively necessary. Dr. Galabin writes: "I believe that one of its (sphygmograph's) successful applications would be to estimate the probable duration of life, by showing how far the vascular system has undergone the changes to which it is subject with advancing years, or which may be the only indication of commencing Bright's disease.

Dr. Handfield Jones has recorded a series of cases in which, without albuminuria or other symptoms of Bright's disease, the spygmograph has sufficed to diagnose the disease, the high-pressure in the pulse being really its pathogomonic symptom. Of the value of the high tension in prognosis he thus writes: "If I were bold enough to be a prophet, I should point to the time when the elder folks, instead of waiting until they have had a stroke of apoplexy or a touch of paralysis, or are laid up with arterio-capillary fibrosis or morbus Brightii, and then hurriedly summoning a physician to do impossibilities, will seek his advice betimes, asking him to supervise their vital functions, regulate their mode of life, and teach them to stay the morbid changes which they know may be silently progressing."

"In a paper published in 1874, Dr. Mahomed tried to show that a chronic condition of high blood-pressure led to arterio-capillary fibrosis. This view he has further elaborated in a paper in the last volume of the *Guy's Hospital Reports*, where he says that the hard pulse—the pulse of high tension—is *the* sign of Bright's disease; and that it should altogether replace albuminuria, for this is only exceptionally present."

"High blood-pressure gives rise to many functional as well as structural disorders. In the paper referred to, Dr. Mahomed described the occurrences of this condition in an acute and transient, as well as in a chronic and permanent, form, as the result of certain blood-poisons, and as an *antecedent* of kidney-disease. He has also demonstrated its existence in pregnancy. and thus indicated the connection between pregnancy and kidney-disease (so-called), and thus with puerperal eclampsia."

"This condition of high blood-pressure, appears to be recognizable early in life, before it has given rise to any structural changes or symptoms, and may be described as depending on the *diathesis*, or less correctly on the *temperament*, of the person. It is a condition which, when once detected, should be closely watched and treated, otherwise it will develop into chronic Bright's disease. * * * * *"

"The possibility of recognizing Bright's disease in its pre-albuminuric stage by means of the sphygmograph leads me to think, with Dr. Handfield Jones, that this instrument has a great future before it. When people become wise enough to pay their doctors for keeping them in health by periodically examining them to see if there be any abnormal change taking place in their bodies, the sphygmograph must come into general use in our profession. How many valuable lives might be prolonged if this suggestion which I have thrown out were acted upon! An indigestion, with increased arterial tension, is, as Dr. Saundby and others have shown, the forerunner of Bright's disease; and this functional stage of the malady is probably quite amenable to treatment. This condition of high arterial tension occurs in those who eat too much nitrogenous food for the exercise they take, and thus overtax their digestive organs and load their blood with imperfectly assimilated food. * * * *"

"One other point of great importance, in reference to albuminuria and arterial tension, which must not be altogether passed over, is the so-called "Albuminuria of Adolescents," which was the subject of a paper by Dr. Moxon in the *Guy's Hospital Reports* for 1878. In those cases of albuminuria which occur in young men from the age of sixteen to twenty-two, the albumen is not always present in the urine, but intermits; it is generally, however, present in the urine at some period of the day, most frequently in that passed after breakfast. These patients are usually languid, listless, complaining of headache, are anæmic, and "generally out of condition;" but after a variable time, under the influence of tonic treatment, the albuminuria usually entirely disappears, and the patient's health is restored. Now, in these cases of albuminuria, the arterial pressure is quite normal, or low rather than high. I have seen several cases of this description amongst the undergraduates of the University and others, and it is of the utmost importance to recognize them clinically; for the prognosis in them is favorable, and the line of treatment to be adopted the very opposite of that which obtains in cases of albuminuria with high arterial tension. I am disposed to agree with Sir William Gull, that the albuminuria in these cases is due to atony of vessels and nerves. He says, that albuminura occurs in young and growing men and boys almost as frequently as spermatorrhœa."

In reference to this subject, Dr. George Johnson maintains, "first, that this latent albuminuria—albuminuria, that is, unassociated with any other evidence of functional disorder or structural disease—may, by a careful inquiry, be traced back, in a very large proportion of cases, to some probable exciting cause; secondly, that the presence of even the smallest trace of albumen in the urine is always pathological, never physiological; and that the neglect of this indication of a pathological condition and tendency, especially such negligence as involves repeated exposure to the exciting cause, may convert a temporary and occasional into a persistent albuminuria, which, sooner or later, though it may be after many years, will result in a fatal disorganization of the kidney."

"The probable causes of such albuminuria are, in Dr. Johnson's opinion, either (1) imperfect recovery from an acute renal attack; or (2) exposure to cold and wet, especially after being overheated and fatigued by prolonged or violent exercise (common in boys at school); or (3) imprudently prolonged cold bathing; or (4) excessive consumption of animal food and of alcoholic stimulants, either separately or combined; or (5) inveterate dyspepsia in persons of strictly temperate habits; or (6) mental anxiety; and, in some cases, a combination of two or more of these causes."

"There can be no doubt that, at the present time, physicians do not regard simply the presence of albumen in the urine as so serious a sign as they would have done ten years ago. The late Dr. Murchison regarded some cases of albuminuria as due to hepatic derangement. In his work on *Disease of the Liver*, second edition, p. 573, he writes as follows: "There are, also, reasons for believing that albuminuria may be induced by hepatic derangement, independently of structural disease of the kidneys. It is now generally acknowledged that albuminuria, even when copious, and in the absence of any acute febrile disorder, does not necessarily indicate renal disease. Very often in these cases the albuminuria is intermittent or remittent, and the albumen has peculiar chemical characters; the previous addition, for example, of a few drops of mineral acid preventing to an unusual extent the subsequent coagulability by heat. Errors in diet are one of the most common causes. In some persons peculiarly constituted, temporary albuminuria is a constant result of certain articles of food, such as uncooked eggs. In several instances, I have known the urine passed at night to contain albumen, often associated with lithates and a high specific gravity, whereas the morning urine was clear, of low specific gravity, and contained no albumen. Again, in certain cases of exophthalmic goitre, the urine at some hours of the day, usually after food, is loaded with albumen, whereas at others it contains none; and this state of matters may last for many months, and then completely disappear. Now it is not contended that, in all these cases, the liver is the organ primarily at fault, but certainly in some there is good reason for believing it to be so, the albuminuria being unattended by any other symptom of renal disease, being variable in quantity and sometimes absent, and the urine being of normal quantity, of high specific gravity, and habitually loaded with lithates, lithic acid, oxalates, and pigments, and there being very often cutaneous eruption, dyspepsia, and other evidence of hepatic derangement. I have met with several instances of this sort, where the patient was subject to severe attacks of what at first seemed to be hepatic colic, but where there was no jaundice, and the paroxysm was followed by a temporary, yet extraordinary, increase of lithates and albumen in the urine. Lastly, so often have I observed albuminuria associated with hepatic disorder, which has disappeared completely and permanently when this has been set to rights, that I have little doubt that we have, in the liver, a cause of albuminuria to which attention has not hitherto been sufficiently directed."

"Before leaving the subject of albuminuria, I would especially invite the general practitioner in medicine to help us to solve the meaning of this intermittent appearance of an abnormal substance in one of the secretions. They have opportunities of watching cases in private from day to day, and of knowing the habits of their patients better than a physician, who is only consulted occasionally. It is of the utmost importance that the question should be settled as to whether these forms of albuminuria are due to removable causes, such as indigestion, etc., or whether, as Dr. Dickinson would have us believe, they represent the initial stages of granular degeneration of the kidney."

5. "On Occipital Headache as a symptom of Uræmia" (Cincinnati Lancet & Clinic), Dr. E. C. Seguin says: "I have recently met with two cases in which occipital headache was so localized and persistent as to give rise to a strong suspicion of organic disease of the cerebellum, and in one of them a positive conclusion was only reached by means of a post mortem examination. These cases both now appear to have been cases of contracted kidney and uræmia."

6. "Recent Studies in Albuminuria," by H. S. Kilbourne, M. D., Asst. Surgeon, U. S. A. (Buffalo Med. and Surg. Journal, Sept. 1880.) Dr. Kilbourne's paper is mainly a *résumé* of the recently expressed views of Saundby, Duke, Johnson, Mahomed and others, but we quote the following observations of Leube, which Dr. Kilbourne thinks "seem to prove the possibility of albuminuria in the physiological state:"

Leube undertook his researches on the garrison of Erlangen and took the necessary precaution to exclude cases of blennorrhagia. Fresh urine was filtered and a certain quantity boiled; the remainder was treated with nitric acid, both being compared with the intact urine on a block tablet. To the urine which showed opacity a small quantity of acetic acid was added to precipitate the deposit. The precipitate was washed and treated with Millou's fluid and also tested with Liq. Potass. Leube examined the night urine of 119 soldiers. The number of observations was 154, which were thus divided: 90 soldiers were examined once, 23 were examined twice on two different days, and six were examined three times at intervals of three days. Of 154 examples of nocturnal urine, only a small quantity was found in 5 cases and in one case in notable proportion.

Examination of the urine secreted during the day, after military exercise, and in the months of June, July and August gave different results. Of 5 soldiers who had shown albumen in the night urine, a larger quantity was found during the day, and albumen was found in 18 soldiers who had not shown any in the night urine. Stated as a percentage, the morning urine was albuminous in 5 of 119 soldiers, i. e., in 4.2 per cent. That of the middle of the day was albuminous in 19 of 119, i. e., 16 per cent. The day urine only was albuminous in 14 of 19, i. e., 11.8 per cent., and, finally, the urine was equally albuminous in 5 of 119, i. e., 4.2 per cent.

The quantity of albumen in the urine most heavily loaded was 38 milligrammes per cent.

At the International Medical Congress, held in Amsterdam, September, 1879, Prof. Semmola in a *résumé* (*British Medical Journal*, September 27, 1879) of his researches in 300 cases of Bright's disease, presented to the medical section, said: The passage of albumen into the urine may take place through the three physiological factors that preside over the renal functions, viz.: *a*, chemical constitution of the blood; *b*, degree of pressure; *c*, condition of the histological elements of the kidney. Accordingly there are three classes of albuminuria, viz.: *a*, dyscrasic albuminuria; *b*, mechanical albuminuria; *c*, mechanical albuminuria produced by irritation, i. e., by histological changes in the kidney.

Bright's disease cannot be placed exclusively under either of these heads. It is a mixed albuminuria. Its complicated eitiological mechanism contains all the three mechanisms of the other classes of albuminuria, and it forms a pathological specialty that has nothing whatever to do with the other classes of this affection. The most characteristic phenomenon of Bright's disease, he thinks, is the defective formation of urea. This would be indicated, not by specific gravity of the urine, but by the weight of urea excreted.

---

UNDER date of May 14th, 1880, Dr. Chas. W. Robbins, of Milwaukee, Wis., makes a brief record of an exceptional case. Mr. M., aged 38, had his left testis removed for malignant disease, in the year 1874. The right gland became similarly affected during the course of the succeeding year, and was subsequently removed by Dr. Marks, of Milwaukee. For the past few months, the cord of the left side, which had not been completely extirpated when the first operation was performed, has been excessively painful, and it was removed on the 13th of May by Dr. Marks, assisted by Dr. Robbins. The patient stated that, since the removal of the second gland, he has regularly had sexual desire, erection and coitus, not apparently differing from that in which he indulged before castration.

